# Breast care session effectiveness on lifestyle modifications and mastectomy clients' lived experience in a tertiary care hospital

**DOI:** 10.6026/973206300213673

**Published:** 2025-10-31

**Authors:** Deepika Kathiravan, Dharani Koorathalvar, Divyalakshmi Kannan, Balasowndarya Ravichandran, Chitra Durai, Deepa Mani, Dhanalakshmi Kuppan, Gopu Govindhasamy, Shankar Shanmugam Rajendran

**Affiliations:** 1 Department of Nursing Research, College of Nursing, Madras Medical College, Affiliated to The Tamil Nadu Dr MGR Medical University, Chennai, India; 2 Department of Surgical Oncology, Affiliated to The Tamil Nadu Dr MGR Medical University, Chennai, India; 3 Department of Child Health Nursing, College of Nursing, Madras Medical College, Affiliated to The Tamil Nadu Dr MGR Medical University, Chennai, India

**Keywords:** Mastectomy clients, Breast care session, lifestyle modification, lived-in experience

## Abstract

A structured breast care session on lifestyle modifications and the lived experiences of post-mastectomy women is of interest. Using a mixed-methods approach, 60 participants were evaluated quantitatively, showing a significant improvement in lifestyle scores (72.85%) after the sessions. Qualitative interviews highlighted themes such as emotional responses, coping mechanisms and family support. Results indicated a strong link between the intervention and improved emotional well-being. Thus, we show that culturally appropriate breast care strategies enhance the recovery and psychosocial health of mastectomy clients.

## Background:

Breast cancer has become the most common malignancy among women in India, with recent statistics revealing over 1,60,000 new cases annually [1]. This increase is largely due to lifestyle changes, urbanization and greater awareness of the disease. Mastectomy remains the predominant treatment for breast cancer, particularly in advanced stages [2]. Unfortunately, late-stage diagnosis and limited screening practices in India contribute to the high number of mastectomies. While mastectomy is a life-saving procedure, it brings significant physical, emotional and social challenges, including body image concerns, loss of femininity and altered self-esteem [3]. The recovery process for mastectomy patients is multifaceted, involving not just physical healing but also emotional and psychological adaptation. Lifestyle modifications, such as healthy nutrition, regular physical activity, stress management and adherence to follow-up care, are critical for both preventing recurrence and improving overall quality of life [4]. However, many patients struggle to implement these changes effectively due to a lack of knowledge, guidance and emotional support. As a result, there is a need for targeted interventions that provide comprehensive care to patient's post-mastectomy [5]. Breast care sessions, structured educational and counseling interventions, have been introduced in various healthcare settings to bridge this gap. These sessions aim to empower patients with knowledge on mastectomy exercises, nutritional guidelines and proper use of prostheses, emotional coping mechanisms and guidance on sexual well-being [6]. They provide a platform for patients to discuss their challenges and learn ways to adapt to their new physical and emotional realities. However, the lived experiences of mastectomy patients, including their body image struggles, emotional responses, stigma and the role of family and community support, have not been fully explored, especially in the Indian context [7]. In India, cultural and societal factors, such as the stigma surrounding breast cancer and mastectomy, further complicate the recovery process. These factors can lead to feelings of isolation, anxiety and fear of being perceived differently by family, friends and society [8]. Furthermore, women in rural areas or those with limited access to healthcare may face additional barriers in receiving adequate post-operative care and counseling. Therefore, understanding the multifaceted challenges that mastectomy patient's face is crucial in developing holistic care strategies tailored to their needs [9]. Therefore, it is of interest to describe how structured interventions can improve the overall quality of life for mastectomy patients and provide them with the tools they need to navigate their post-surgery journey with greater confidence and well-being.

## Methodology:

This study employed a mixed-methods approach using an Exploratory Sequential design, which combined both qualitative and quantitative data. The qualitative data provided detailed insights into the personal experiences and challenges faced by mastectomy patients. In contrast, the quantitative data offered a broader understanding of the impact of breast care sessions on lifestyle modifications among these patients. The qualitative part of the study utilized a Phenomenological method, which focuses on exploring and capturing the subjective lived experiences of mastectomy clients. This method aimed to understand and describe phenomena as individuals perceive them through their lived experiences. In-depth, semi-structured interviews were conducted to collect personal narratives from mastectomy patients, focusing on their challenges and experiences after surgery. The quantitative part of the study used a Quasi-Experimental, one-group pretest-posttest control Group Design to assess the impact of breast care sessions on lifestyle modification among mastectomy patients. The pre-test was conducted before the intervention and the post-test was conducted after the intervention to evaluate changes in lifestyle modifications. The study design followed a structured process with an experimental group undergoing the intervention, which consisted of breast care sessions. The pre-test was conducted to assess the initial level of lifestyle modification. After the intervention (breast care session), a post-test was conducted to evaluate the impact of the session on lifestyle changes.

The study was conducted in the Surgical Oncology Outpatient Department (OPD) at Rajiv Gandhi Government General Hospital (RGGGH), Chennai. This department was chosen as the setting to provide a comprehensive view of the impact of breast care sessions among mastectomy patients, allowing the study to examine various dimensions of lifestyle modification strategies and quality of care. Data collection was carried out over a 3-week period. The target population for the study consisted of mastectomy patients attending the Surgical Oncology OPD at RGGGH, Chennai. The accessible population included those patients who were willing to participate and available during the study period. The sample was selected using purposive and convenient sampling techniques. For the qualitative part of the study, 7 mastectomy patients who experienced physical mobility challenges were selected. For the quantitative part, 50 mastectomy patients attending the Surgical Oncology OPD were included in the study, as per the sample size determination based on a prior study. Quantitative sample size was calculated to be 60 ± 10. The study utilized non-probability purposive sampling for the qualitative part and non-probability convenient sampling for the quantitative part. This ensured that the sample was rich in information relevant to the study objectives, providing a deep understanding of the research topic. Inclusion criteria for the qualitative part included individuals who were willing to share their personal lived experiences and challenges, while the quantitative part focused on post-mastectomy patients who were attending the OPD for follow-up care and exhibited lifestyle challenges as a result of mastectomy. Exclusion criteria included patients with severe physical, sensory, or cognitive impairments, those with other neurological or psychiatric disorders and patients currently enrolled in other research studies or with a history of non-compliance. The research variables included the independent variable of breast care sessions (which involved educational tools like videos, pamphlets, models, booklets and health teaching) and the dependent variable of lifestyle modification, measured through a lifestyle modification questionnaire. For the qualitative part, in-depth interviews were conducted using a semi-structured interview schedule to explore the lived experiences of mastectomy clients. For the quantitative part, a two-section questionnaire was used. Section A covered socio-demographic characteristics (such as age, gender, marital status and family structure) and Section B included a Lifestyle Modifications Questionnaire, which used a 3-point Likert scale (ranging from 0 to 2) to assess the patients' nutrition, physical activity and sexual health. The higher the score, the greater the improvement in healthy lifestyle behaviors.

The Lifestyle Modification Score was interpreted as follows:

[1] 0-23: High-risk, requiring multidisciplinary intervention.

[2] 24-33: Needs improvement, with personalized lifestyle modification plans.

[3] 34-43: Moderate adaptation, recommending targeted education and support.

[4] 44-54: Excellent lifestyle adaptation, with minimal intervention needed.

Content validity of the tools used in the study was assessed by experts in surgical oncology and medical surgical nursing, ensuring that the instruments were appropriate and relevant to the research objectives. Reliability was tested using the Cronbach's Alpha method, with a reliability score of α = 0.82, indicating high reliability. Before conducting the main study, a pilot study was conducted with two mastectomy patients selected through purposive sampling. Their experiences were recorded and analyzed using thematic content analysis. The pilot study tested the feasibility of the data collection procedures and confirmed that the lifestyle modification scores among participants improved after the intervention, indicating the study's feasibility. The data collection procedure included obtaining a valid clearance letter from the Dean and Head of the Surgical Oncology Department at RGGGH. Participants were informed about the study's purpose and gave their informed consent before participation. For the qualitative part, participants were selected using purposive sampling and interviews were conducted to gather personal insights into their experiences. For the quantitative part, participants completed the lifestyle modification questionnaire and breast care sessions were provided to the experimental group. Post-intervention lifestyle modification scores were then measured. The intervention protocol for the breast care session included a 30-minute session using videos, pamphlets, models and booklets. The session aimed to educate patients on post-mastectomy exercises, dietary needs, emotional stabilization and sexual health. The session emphasized the importance of lifestyle modifications, including physical activity and proper nutrition, to improve overall health and well-being. For data analysis, both descriptive and inferential statistics were used. Qualitative data were analyzed using thematic content analysis, identifying recurring themes and patterns in the experiences of mastectomy patients. Quantitative data were analyzed using descriptive statistics (mean, median, standard deviation) and inferential statistics (paired t-test and chi-square test) to evaluate the significance of the intervention and its association with demographic variables. The results were presented in tables and graphs and statistical significance was determined at p < 0.05. In summary, this study used a combination of qualitative and quantitative methods to explore the impact of breast care sessions on lifestyle modifications among mastectomy patients, aiming to provide valuable insights into the physical, emotional and lifestyle changes that these patients experience during their recovery.

## Results:

Table 1 (see PDF) presents the demographic characteristics of the study participants. The majority of women were above 50 years of age (56.67%), with 35% in the 41-50 years age group. Most participants had primary education (71.67%) and the largest portion of the family income ranged from 7000-8000 rupees (45%). The marital status data shows that 93.33% of participants were married. Regarding dietary habits, a mixed diet was most common (48.33%) and the majority resided in urban areas (60%). Most participants were homemakers (81.67%) and a significant number had no history of breast disease (91.67%). Finally, most participants had two children (55%). With regard to the distribution of the age group of mastectomy survivors attending the study are about 56.67% of clients are above 50 years, 35% of clients are 41-50 years, 5% of about 31-40 years and 3.33% is 21-30 years. In regard to educational status, 71.67% are having their Primary education, 18.33% is about secondary education, 3.33% is about higher education and 6.67% is about others. In regards to family income, 26.67% is having 5000-6000, 45% is about 7000-8000, 11.66% is having 9000-10000 and 16.67% is having above 10000. In regard to the marital status, 93.33% is married and 6.67% is not married. In regards to Dietary habits, 8.34% is vegetarian 43.33% is non-vegetarian and 48.33% is taking mixed diet. In regards to Habitat, 60% of them are in urban and 40% of them are in rural area. In regards to Occupation, Self-employment is about 11.66%, Homemaker is 81.67% and some private jobs are about 6.67%. In regards to the history of breast diseases, 8.33% is having history and 91.67% is having no history. In regards to the number of children it is about 8.34% of them is having 1 child, 55% of them is having 2 children and 33.33% is having 3 or more children and 3.33% of them is having no children. The distribution of age among the mastectomy survivors attending the study shows that 56.67% of participants are above 50 years of age, 35% are between 41-50 years, 5% are between 31-40 years and 3.33% are between 21-30 year. In terms of educational status, 71.67% of the participants have completed primary education, 18.33% have secondary education, 3.33% have higher education and 6.67% have other forms of education. Regarding family income, 26.67% of participants earn between 5000-6000 rupees, 45% earn between 7000-8000 rupees, 11.66% earn between 9000-10000 rupees and 16.67% earn above 10000 rupees. In terms of marital status, 93.33% of participants are married, while 6.67% are unmarried. As for dietary habits, 8.34% of participants are vegetarian, 43.33% are non-vegetarian and 48.33% follow a mixed diet. Regarding habitat, 60% live in urban areas and 40% reside in rural areas. In terms of occupation, 11.66% are self-employed, 81.67% are homemakers and 6.67% work in private jobs. Regarding the history of breast diseases, 8.33% of participants have a history of breast disease, while 91.67% have no such history. In terms of the number of children, 8.34% have one child, 55% have two children, 33.33% have three or more children and 3.33% have no children.

Table 2 (see PDF) presents the demographic variables of the mastectomy clients who participated in the study. Regarding age at menarche, 63.33% of women experienced menarche between 12-14 years, 28.34% had menarche after the age of 14 and 8.33% had it before the age of 11. In terms of history of marriage, 53.33% of participants married between the ages of 21-30 years, while 46.67% married before the age of 20. No participants reported marrying between the ages of 31-40 years or after the age of 40. For menstrual history, 25% of the participants reported having a regular menstrual cycle, 18.33% had an irregular cycle and 56.67% had attained menopause. Regarding the history of menopause, 21.67% of participants reached menopause before the age of 40, 38.33% reached menopause between the ages of 40-45 and 40% had not yet attained menopause. Table 3 (see PDF) presents the responses to a series of statements regarding lifestyle modifications post-mastectomy. The questionnaire includes 18 statements that assess various aspects of recovery, such as physical health (e.g., pain, arm mobility), emotional well-being (e.g., body image, emotional distress), health habits (e.g., diet, exercise) and support systems (e.g., psychological support, follow-up care). The table highlights the percentage distribution of responses across three categories: "No" (0), "Sometimes" (1) and "Yes" (2). Notably, the majority of respondents report not smoking or drinking alcohol and a significant proportion is engaged in regular physical activity or walking. A large percentage feels discomfort with their body image post-mastectomy, while many express interest in being referred to a support group or receiving additional education on lifestyle changes. Table 4 (see PDF) summarizes the mean and standard deviation scores for different domains of lifestyle modifications, reflecting the overall health, emotional and psychological adjustments post-mastectomy. The highest mean score is in the "Lifestyle & Habits" domain (5.83), indicating that most patients engage in lifestyle modifications like exercise and diet changes. The "Physical Recovery" domain has a mean of 3.73, showing moderate recovery. Emotional and psychological well-being, as well as follow-up care, also score relatively well with means of 4.30 and 4.18, respectively. The domains with lower scores include "Sexual Health & Intimacy" (1.95) and "Prosthesis & Reconstruction" (0.40), suggesting that these areas might require more focus or education in future interventions. The total mean score of 20.40 indicates that overall, patients are moderately engaging in lifestyle changes post-mastectomy. As for the history of other cancers, 5% of participants had a history of another type of cancer, while 95% had no such history. When asked about a family history of breast cancer, 98.33% reported no family history, while 1.67% had a family history, with no specific types of breast cancer identified. In terms of co-morbid illnesses, 18.33% of participants reported having a co-morbid illness, 56.67% did not and 25% did not provide specific details on the illness. Regarding high-risk pregnancies, only 1.67% reported a history of a high-risk pregnancy, while 98.33% did not. Regarding the use of contraceptive pills, 10% of participants had used contraceptive pills, while 90% had not. As for breastfeeding history, the majority, 91.67%, had breastfed, while 8.33% had not. Finally, concerning the timing of mastectomy, 25% of participants had undergone mastectomy within the last year, 60% had it 1-5 years ago and 15% had their mastectomy more than 5 years ago. This table provides a comprehensive overview of the various demographic factors related to the mastectomy clients in the study. Table 5 (see PDF) shows each domain wise percentage of Life style Modification score. They are having more percentage of score for the domain lifestyle & habits (72.88%) and they are having less percentage of score for the domain (20.00%). Table 6 (see PDF) shows the percentage level of lifestyle modification score among women. In general, 38.33% of them are having low level of score, 61.67% of them are having moderate level of score, none of them having high level of score.

## Level of lifestyle modification score interpretation:

Min = 0 Max = 2 Total items = 18 Total score = 36

Above table shows the association between level of lifestyle modification score and demographic variables. More income and urban mastectomy women are having more moderate lifestyle modification score. Statistical significance was calculated using chi square test. Table 6 (see PDF) shows the association between level of lifestyle modification score and demographic variables. Menopause attained women are having more moderate lifestyle modification score. Statistical significance was calculated using chi square test. The association between lifestyle modification scores and various demographic variables was analyzed in the present study, with results detailed in Tables 7 (see PDF), 8 (see PDF), and 9 (see PDF). Table 7 (see PDF) provides further insights into the demographic and health-related factors influencing lifestyle modifications. Table 8 (see PDF) presents the relationship between lifestyle modification scores and demographic variables such as age, education, family income, marital status, and others. For example, a significant association was found between family income and lifestyle modification (p = 0.01), as well as between habitat (urban vs. rural) and lifestyle modification (p = 0.01). In Table 9 (see PDF), similar associations were explored concerning age at menarche, history of menopause, and other health conditions. For instance, a significant association was observed between menstrual history (regular vs. irregular cycles) and lifestyle modification (p = 0.01). Table 7 (see PDF) provides further insights into the demographic and health-related factors influencing lifestyle modifications.

## Discussion:

The findings of this study are consistent with several previous studies exploring the experiences of post-mastectomy women and their lifestyle modifications. Davies *et al.* (2017) [10] conducted a phenomenological research study involving 10 women and found that these women faced ongoing challenges, particularly when they saw their mastectomy scars for the first time. The study indicated that these challenges were not adequately addressed by healthcare professionals, suggesting that nurses and other healthcare providers need to develop a better understanding of the difficulties perceived by women following mastectomy. Patiya *et al.* (2023) [11] conducted a similar qualitative phenomenological study with 14 post-mastectomy women to explore their lived experiences. The study revealed five key themes: body image changes, physical impacts, emotional impacts, treatment impacts and coping and support sources. The study concluded that counseling sessions should be conducted both before and after mastectomy by professional counselors to address the multifaceted needs of these women. Ahn *et al.* (2023) [12] explored the experiences of 15 women who underwent immediate breast reconstruction due to breast cancer. Their study concluded that breast reconstruction helped women regain their sense of body image, contributing to their psychological recovery and enabling them to return to daily life. This finding underscores the importance of culturally appropriate counseling and interventions to improve women's perceptions of their body image post-mastectomy. Khubchandani *et al.* (2025) [13] explored cultural and community-related themes after mastectomy. Their study, which involved 20 women from diverse ethnic backgrounds, found that cancer stigma, privacy around breasts and the role of family support significantly influenced the lived experiences of these women. This study highlighted how cultural and societal expectations intersected with their experiences, affecting their knowledge of family history and the support they received during cancer treatment. The above studies demonstrate that the post-mastectomy experience is complex and multifaceted, involving emotional distress, body image alterations, cultural influences and the critical role of support systems. These findings emphasize the recurring theme of the need for holistic, empathetic and culturally sensitive care to address the physical, emotional and social aspects of recovery.

Bieyabanie *et al.* (2020) [14] conducted a cross-sectional study involving 100 mastectomy women in Tabriz, Iran. The study found that factors like age, spouse's educational attainment and self-efficacy were significant predictors of health-promoting lifestyle behaviors. This highlights the importance of self-efficacy in adopting a health-promoting lifestyle post-mastectomy. Hsia *et al.* (2024) [15] conducted a longitudinal study on 53 female breast cancer survivors to examine the factors influencing health-promoting lifestyles. The study found that self-efficacy; psychological well-being and responsible health practices were significant predictors of health-promoting behaviors, suggesting that improving self-efficacy is crucial for enhancing lifestyle modifications. Shamloo *et al.* (2023) [16] explored the experiences of mastectomy clients, their husbands, oncologists and healthcare professionals. The study identified four dimensions: physical, self-concept, role-playing and interdependence. The results showed that mastectomy patients faced various physical and mental challenges, often leading to role disruptions. The study recommended strategies to improve the adaptation of these patients, reinforcing the need for comprehensive support. Rashwan *et al.* (2024) [17] conducted a quasi-experimental study in Egypt to assess the effects of bundling seated exercises and psychoeducational rehabilitation on post-mastectomy women. The study found that these interventions helped reduce cancer-related fatigue and improve coping skills, demonstrating that non-pharmacological methods can be effective in managing post-mastectomy challenges. Jariwala *et al.* (2021) [18] conducted a descriptive study on breast cancer survivors in Delhi and found that 49% of survivors had significant arm and shoulder problems, including swelling. This highlights the need for focused interventions to address physical impairments that affect the daily life of mastectomy patients. These studies collectively demonstrate the complex and varied challenges that post-mastectomy women face, including physical, emotional and psychological impacts. They highlight the importance of providing comprehensive, personalized care that addresses both the physical and emotional aspects of recovery. The recurring theme across these studies is the need for culturally sensitive, holistic support systems to improve the overall quality of life for mastectomy patients.

## Conclusion:

The demographic data of mastectomy clients reveals a diverse group in terms of age, marriage history, menstrual history and health conditions. The majority of participants experienced menarche between 12-14 years, married between 21-30 years and most had a history of breastfeeding. Thus, we show the importance of personalized care and support for mastectomy patients based on their varied backgrounds and health experiences.

## References

[1] Mehrotra R (2022). World J Clin Oncol..

[2] Francies FZ (2020). Am J Cancer Res..

[3] Rose MM (2024). J Midlife Health..

[4] Hamer J (2017). CMAJ..

[5] King R (2024). J Cancer Surviv..

[6] Mishra A (2023). Breast Cancer (Auckl)..

[7] Koçan S (2016). J Breast Health..

[8] Merino M (2024). Healthcare..

[9] Mansour R (2024). Cancers (Basel)..

[10] Davies CC (2017). Cancer Nurs..

[11] Patiyal N (2023). J Educ Health Promot..

[12] Ahn J (2023). Asia Pac J Oncol Nurs..

[13] Khubchandani JA (2025). Ann Surg Oncol..

[14] Hamed Bieyabanie M (2020). Asian Pac J Cancer Prev..

[15] Hsia HH (2024). Semin Oncol Nurs..

[16] Shamloo MBB (2023). Int J Palliat Nurs..

[17] Rashwan ZI (2024). BMC Womens Health..

[18] Jariwala P (2021). Indian J Cancer..

